# Accelerated retinal ageing and multimorbidity in middle-aged and older adults

**DOI:** 10.1007/s11357-025-01581-1

**Published:** 2025-03-04

**Authors:** Ruiye Chen, Xiaomin Zeng, Wenyi Hu, Deepak Jeyarajan, Zhen Yu, Wei Wang, Zongyuan Ge, Xianwen Shang, Mingguang He, Zhuoting Zhu

**Affiliations:** 1 Centre for Eye Research Australia; Ophthalmology, University of Melbourne, Melbourne, Australia; 2 Department of Surgery, University of Melbourne, Melbourne, Australia; 3 Department of Ophthalmology, Guangdong Academy of Medical Sciences, Guangdong Provincial People’s Hospital, Guangzhou, China; 4 Monash School of Medicine, Faculty of Medicine, Nursing and Health Sciences, Monash, University, Melbourne, Australia; 5 Faculty of IT, Monash University, Melbourne, Australia; 6 Monash Medical AI, Monash University, Melbourne, Australia; 7 State Key Laboratory of Ophthalmology, Zhongshan Ophthalmic Center, Sun Yat-Sen University, Guangzhou, China; 8 School of Optometry, The Hong Kong Polytechnic University, Kowloon, Hong Kong China; 9 Research Centre for SHARP Vision (RCSV), The Hong Kong Polytechnic University, Kowloon, Hong Kong, China; 10 Centre for Eye and Vision Research (CEVR), 17W Hong Kong Science Park, Hong Kong, China

**Keywords:** Retinal age, Multimorbidity, Association, Biological age

## Abstract

The aim of this study is to investigate the association between retinal age gap and multimorbidity. Retinal age gap was calculated based on a previously developed deep learning model for 45,436 participants. The number of age-related conditions reported at baseline was summed and categorized as zero, one, or at least two conditions at baseline (multimorbidity). Incident multimorbidity was defined as having two or more age-related diseases onset during the follow-up period. Linear regressions were fit to examine the associations of disease numbers at baseline with retinal age gaps. Cox proportional hazard regression models were used to examine associations of retinal age gaps with the incidence of multimorbidity. In the fully adjusted model, those with multimorbidity and one disease both showed significant increases in retinal age gaps at baseline compared to participants with zero disease number (*β* = 0.254, 95% CI 0.154, 0.354; P < 0.001; *β* = 0.203, 95% CI 0.116, 0.291; *P* < 0.001; respectively). After a median follow-up period of 11.38 (IQR, 11.26–11.53; range, 0.02–11.81) years, a total of 3607 (17.29%) participants had incident multimorbidity. Each 5-year increase in retinal age gap at baseline was independently associated with an 8% increase in the risk of multimorbidity (HR = 1.08, 95% CI 1.02, 1.14, *P* = 0.008). Our study demonstrated that an increase of retinal age gap was independently associated with a greater risk of incident multimorbidity. By recognizing deviations from normal aging, we can identify individuals at higher risk of developing multimorbidity. This early identification facilitates patients’ self-management and personalized interventions before disease onset.

**Supplementary Information:**

The online version contains supplementary material available at 10.1007/s11357-025-01581-1.

## Introduction

Multimorbidity, defined as the co-occurrence of two or more chronic conditions [1], affects up to 95% of populations aged 65 years and older [2]. The World Health Organization (WHO) projects that the population aged 65 years and older will increase dramatically to 1.5 billion between 2019 and 2050 [3]. With the ageing of the global population, the health burden caused by multimorbidity is expected to increase significantly, positioning it as a prioritized agenda for policymakers and healthcare providers.

The concept of biological age has been recently proposed as a precise index to quantify the ageing process [4]. Unlike chronological age, which does not reflect individual variation, biological age reflects progressive structural, physiological, and functional changes and intra‐individual difference. This makes biological age a valuable tool for identifying individuals at greater risk for age-related diseases and tailoring personalized interventions to promote healthy aging. A series of proposed ageing biomarkers were developed such as telomere length, epigenetic clock, and brain age [5–7].

Retinal age has emerged as a non-invasive, accessible, and cost-effective biomarker of ageing [8, 9]. The retina has been considered as a window to the body, providing unique insights into systemic health [10, 11]. Our previous study utilized deep learning to develop a retinal age model using retinal imaging from a diverse population [9]. Then, a retinal age gap, the deviation between the predicted retinal age and chronological age, was considered to represent accelerated aging. A larger retinal age gap has been linked to a higher risk of various chronic conditions, including cardiovascular diseases [12–14], neurodegenerative disorders [15], and kidney diseases [16]. Moreover, the ease and non-invasive nature of retinal imaging position retinal age as a feasible tool in real world settings.

As multimorbidity is not a single disease affecting one system; rather, it involves the concurrent deterioration of multiple organ systems, much like the ageing process [17]. Multimorbidity could be potentially considered as a transitional phase between ageing and death, reflecting a middle stage with shared underlying mechanisms [18]. Despite this connection, limited research has investigated the association between biological age biomarkers and multimorbidity. This gap in understanding presents an opportunity to explore whether retinal age could serve as a potential indicator for identifying individuals at risk of multimorbidity. Additionally, this approach can provide insights into better management strategies for multimorbidity. Therefore, we aim to study the association between retinal age gap and the risk of multimorbidity in a prospective cohort of UK biobank.

## Methods

### Study design and population

This study utilized data from the UK Biobank, a large, prospective, population-based cohort comprising over 500,000 participants recruited between 2006 and 2010. Participants aged 40–69 years underwent baseline assessments including comprehensive lifestyle and health questionnaires, physical measurements, and sample collection at 22 assessment centres located across England, Scotland, and Wales. Medical event information was obtained through data linkage to hospital admission records and death registers. The study design and protocols have been detailed elsewhere [19]. Comprehensive ophthalmic examinations were introduced in late 2009 at six assessment centres for baseline measures [20]. Fundus photography (Topcon 3D OCT 1000 Mk2, Topcon Corp, Tokyo, Japan) was used to acquire 45-degree macular-centred non-mydriatic retinal fundus images for each eye.

### Ethics approval and consent to participate

UK Biobank has ethic approval from the North West Multi-centre Research Ethics Committee (MREC) as a Research Tissue Bank (RTB) approval (Reference number: 11/NW/0382). This RTB approval was granted initially in 2011 and it is renewal on a 5-yearly cycle. All participants provided informed consent at the time of recruitment, acknowledging that their biological samples and health-related data would be collected, securely stored, and used for approved research purposes. Access to the UK Biobank data was obtained through the Biobank consortium under Application Number 94372. All data were provided in anonymized form, and all analyses were conducted in accordance with the Biobank’s policies and procedures to ensure participant confidentiality.

### Retinal age gap

A deep learning (DL) model for age prediction was developed and validated using a large dataset of retinal fundus images [9]. The model was trained on a diverse healthy cohort to ensure generalizability and robustness of the age estimation. The DL model demonstrated a strong predictive capability with a mean absolute error (MAE) of 3.03 years between retina-predicted age and chronological age, reflecting its accuracy in estimating age from retinal images. Our previous attention map study highlighted those areas around the vessels in the retinal imaging contributed most to the age estimation.

We subsequently applied this DL model to assess the retina-predicted age for the remaining UK biobank participants with available fundus images. An automated image quality assessment process was embedded in the algorithm using a grading model trained on the EyePACS-Q dataset [21]. All images undergoing the quality check were classified as either “reject” or “good/usable.” Only images labeled as “good/usable” were fed into the age estimation algorithm to generate the calculated retinal age. Fundus images of the right eye were used for the age prediction; if the image of the right eye was not available, a fundus image of the left eye was used. The retinal age gap (retina-predicted age minus chronological age) was calculated for a total of 45,436 participants. A larger retinal age gap indicates an accelerated ageing process. Moreover, we divided the participants into three groups of patients who had a predicted retinal age >3 years smaller than the chronological age (>3 years younger), retinal age within a range of 3 years from their chronological age (within ± 3 years), and retinal age >3 years greater than the chronological age (> 3 years older). We chose the cut-off value at 3 years due to the MAE of 3.03 to minimize the impacts of systematic bias in age prediction.

### Multimorbidity

Baseline conditions of major age-related chronic diseases were attained from self-reported information about whether the participants had ever been diagnosed by a doctor (Supplementary Table 1). Additional baseline cases were identified through inpatient hospital records. The International Classification of Diseases 10^th^ Revision (ICD-10) used to define the full list of diseases applied in this study was listed in Supplementary Table 2. The major categories we included are cardiovascular diseases (heart failure, hypertension, coronary artery disease, atrial fibrillation, stroke, peripheral artery disease), metabolic diseases (diabetes, hyperlipoidemia), neurological diseases such as dementia, and other miscellaneous diseases. The number of age-related conditions reported at baseline was summed and categorised as zero, one, or at least two conditions at baseline (multimorbidity). Incident cases of individual diseases from participants without reported major age-related diseases were further identified. Date of each disease onset was defined as the earliest recorded date available. Incident multimorbidity was defined as having two or more age-related diseases onset during the follow-up period. The follow-up period was defined as the time of occurrence of multimorbidity or loss to follow-up or death, whichever came earliest.

### Covariates

Potential confounding factors were adjusted in the current analysis. These include age, gender (male/female), ethnicity (white/others), Townsend deprivation index (TDI), body mass index (BMI), education (college/university or others), physical activity (meeting moderate/vigorous/walking recommendation or not), smoking status (never or ex/current smoker), alcohol consumption (never or ex/current drinker), and genetic risk score (GRS) for longevity. Information on age, sex, ethnicity, education, alcohol consumption, physical activity, and smoking status was collected through touch-screen questionnaires. TDI was derived from participant postcodes as an area-specific measure of socioeconomic deprivation (a higher TDI represents lower socioeconomic status) [22]. BMI was calculated as body weight in kilograms divided by height squared in meters. Genetic risk score (GRS) for longevity was computed using 78 single-nucleotide polymorphisms, with a higher score representing a higher genetic susceptibility to longevity [23]. GRS for longevity was included to adjust for the genetic predisposition to experiencing a lower risk of age-related diseases.

### Statistical analysis

Descriptive statistics, including mean (standard deviation [SD]) or median (interquartile range [IQR] if skewed) for continuous variables and numbers (proportions) for categorical variables, were used to present participant characteristics stratified by three disease number categories. One-way ANOVA and Pearson’s *χ*^2^ tests were employed to compare the differences between groups for continuous and categorical variables, respectively. Age- and sex-adjusted models were performed to assess the potential associations between baseline characteristics and retinal age gaps. Linear regressions were fit to examine the associations of disease numbers at baseline with retinal age gaps. Cox proportional hazard regression models were used to examine associations of retinal age gaps with the incidence of multimorbidity. Three models were tested, with model 1 adjusted for age and gender; model 2 adjusted for age, gender and ethnicity, TDI, education, physical activity, smoking status, alcohol drinking status, BMI; model 3 adjusted for covariates in model 2 and longevity GRS. Regression coefficients (*β*) or hazard ratios (HR) and the corresponding 95% confidence intervals (CI) were calculated for linear regression and cox regression, respectively. To investigate potential nonlinear associations between retinal age gap and incident multimorbidity, a restricted cubic spline analysis was fitted to investigate the nonlinear relationship. Potential interaction terms with retinal age gap were performed for each confounding factor. A two-sided *p*-value of <0.05 indicated statistical significance. All statistical analyses were performed using R (version 3.3.0; R Foundation for Statistical Computing) and Stata (version 13; StataCorp).

## Results

### Study population

Baseline characteristics of 45,436 participants overall and stratified by baseline disease number categories are shown in Table 1. Participants had a mean age of 55.67 ± 8.21 (SD, standard deviation) years, and 55.35% were female. There were significant differences between disease number categories for all covariates (all *P*s < 0.001). Age-related disease numbers identified at baseline ranged from 0 to 11, with 20,857 reported none of the disease and 13,372 participants reported one and 11,207 reported two or more. Compared with participants with zero disease, participants with multimorbidity at baseline tended to be older, male, of white ethnicity, to have higher TDI and lower education level, less active physically, more smokers, and less alcohol consumers, and to have higher body mass index, a lower longevity GRS.

**Table 1:** Baseline characteristics stratified by disease number groups at baseline

Baseline characteristics	Overall	Disease number category			*p* value
		0	1	≥2	
Number of participants	45,436	20,857(45.90)	13,372 (29.43)	11,207 (24.67)	
Age, years, (mean (SD))	55.67 (8.21)	53.04 (7.97)	56.22 (7.96)	59.91 (6.96)	**<0.001**
Sex, *n* (%)					**<0.001**
Female	25,151 (55.35)	11,938 (57.24)	7519 (56.23)	5694 (50.81)	
Male	20,285 (44.65)	8919 (42.76)	5853 (43.77)	5513 (49.19)	
Ethnicity, *n* (%)					**<0.001**
White	41,592 (91.54)	18,975 (90.98)	12,296 (91.95)	10,321 (92.09)	
Others	3844 (8.46)	1882 (9.02)	1076 (8.05)	886 (7.91)	
Townsend, mean ± SD	−1.07 (2.96)	−1.10 (2.92)	−1.11 (2.96)	−0.95 (3.04)	**<0.001**
Education, *n* (%)					**<0.001**
College/university	16,687 (36.73)	8543 (40.96)	4897 (36.62)	3247 (28.97)	
Others	28,749 (63.27)	12314 (59.04)	8475 (63.38)	7960 (71.03)	
Meeting moderate/vigorous/walking recommendation, *n* (%)					**<0.001**
No	6442 (17.16)	2805 (16.01)	1834 (16.72)	1803 (19.92)	
Yes	31097 (82.84)	14,714 (83.99)	9135 (83.28)	7248 (80.08)	
Smoking status, *n* (%)					**<0.001**
Never	25,812 (57.12)	12,535 (60.41)	7592 (57.03)	5685 (51.10)	
Ex/current	19,377 (42.88)	8215 (39.59)	5721 (42.97)	5441 (48.90)	
Alcohol drinking status, *n* (%)					**<0.001**
Never	2063 (4.56)	891 (4.29)	567 (4.25)	605 (5.42)	
Ex/current	43,213 (95.44)	19,891 (95.71)	12,768 (95.75)	10,554 (94.58)	
Body mass index, kg/m^2^ (mean (SD))	27.21 (4.72)	26.28 (4.24)	27.26 (4.64)	28.90 (5.17)	**<0.001**
Longevity genetic risk scores (mean (SD))	0.50 (0.05)	0.50 (0.05)	0.50 (0.05)	0.49 (0.05)	**<0.001**

*SD* standard deviation

Retinal age gap in the study population followed a nearly normal distribution (Supplementary Figure 1). The mean (SD) of retinal age gap is 0.54 (4.06) years. Age- and sex-adjusted models showed that older age, male sex, non-White ethnicity, meeting exercise recommendations, and higher longevity genetic risk scores were associated with lower retinal age gaps while higher Townsend deprivation, smoking, alcohol consumption, and BMI was significantly associated with larger retinal age gaps (all *P* value < 0.05, Supplementary Table 3).

### Disease numbers and retinal age gap at baseline

The associations between disease numbers and retinal age gap are shown in Table 2. After adjusting for age and gender, disease numbers had significantly associations with retinal age gaps (model 1: *β* = 0.093, 95% CI 0.064, 0.122, *P* < 0.001). When participants were categorized into different disease number groups, compared to participants with zero disease number, those with multimorbidity and one disease both showed significant increases in retinal age gaps (model 1: *β* = 0.306, 95% CI 0.219, 0.392; *P* < 0.001; *β* = 0.249, 95% CI = 0.171, 0.327; *P* < 0.001; respectively). These findings remained significant after comprehensive adjustments for covariates (model 3: *β* = 0.254, 95% CI 0.154, 0.354; *P* < 0.001; *β* = 0.203, 95% CI 0.116, 0.291; *P* < 0.001; respectively).

**Table 2:** Association between retinal age gap and disease numbers diagnosed at baseline

	Model 1		Model 2		Model 3	
	Beta (95% CI)	*p* value	Beta (95% CI)	*p* value	Beta (95% CI)	*p* value
Disease numbers	0.093 (0.064, 0.122)	**<0.001**	0.075 (0.042, 0.108)	**<0.001**	0.071 (0.037, 0.105)	**<0.001**
Disease number category						
0	Reference		Reference		Reference	
1	0.249 (0.171, 0.327)	**<0.001**	0.200 (0.114, 0.286)	**<0.001**	0.203 (0.116, 0.291)	**<0.001**
≥2	0.306 (0.219, 0.392)	**<0.001**	0.263 (0.166, 0.361)	**<0.001**	0.254 (0.154, 0.354)	**<0.001**

*Beta* regression coefficient, *CI* confident interval

Model 1 adjusted for age and sex

Model 2 adjusted for covariates in model 1 and ethnicity, Townsend, education, physical activity, smoking status, alcohol drinking status, body mass index

Model 3 adjusted for covariates in model 2 and longevity genetic risk scores (GRS)

### Retinal age gap and multimorbidity

After a median follow-up period of 11.38 (IQR, 11.26–11.53; range, 0.02-–11.81) years, a total of 3607 (17.29%) participants had incident multimorbidity. As shown in Table 3, compared with those who did not experience the outcome, participants with incident multimorbidity tended to be older, male, of white ethnicity, and have lower TDI and education level, smokers, to have higher body mass index, and with lower longevity GRS (all *P*s < 0.001).

**Table 3:** Baseline characteristic of participants without reported diseases of interests stratified by incident multimorbidity

Baseline characteristics	Incident multimorbidity	Non-incident multimorbidity	*p* value
Number of participants	3607	17,250	
Age, years, (mean (SD))	57.67 (7.56)	52.08 (7.71)	**<0.001**
Sex, *n* (%)			
Female	1876 (52.01)	10,062 (58.33)	**<0.001**
Male	1731 (47.99)	7188 (41.67)	
Ethnicity, *n* (%)			
White	3337 (92.51)	15,638 (90.66)	**<0.001**
Others	270 (7.49)	1612 (9.34)	
Townsend, mean ± SD	−1.27 (2.85)	−1.06 (2.94)	**<0.001**
Education, *n* (%)			
College/university	1196 (33.16)	7347 (42.59)	**<0.001**
Others	2411 (66.84)	9903 (57.41)	
Meeting moderate/vigorous/walking recommendation, *n* (%)			
No	471 (16.24)	2334 (15.97)	0.732
Yes	2429 (83.76)	12,285 (84.03)	
Smoking status, *n* (%)			
Never	1958 (54.57)	10,577 (61.63)	**<0.001**
Ex/current	1630 (45.43)	6585 (38.37)	
Alcohol drinking status, *n* (%)			
Never	152 (4.24)	739 (4.30)	0.901
Ex/current	3437 (95.76)	16,454 (95.70)	
Body mass index, kg/m^2^ (mean (SD))	27.33 (4.64)	26.06 (4.12)	**<0.001**
Longevity genetic risk scores (mean (SD))	0.49 (0.05)	0.50 (0.05)	**<0.001**

*SD* standard deviation

After adjusting for age and sex, each 5-year increase in retinal age gap was independently associated with a 12% increase in the risk of multimorbidity (model 1: HR = 1.12, 95% CI =1.07, 1.18; *P* < 0.001), as shown in Table 4. This association remained significant after further adjustments (model 2: HR = 1.09, 95% CI 1.03, 1.15, *P* = 0.003; model 3: HR = 1.08, 95% CI 1.02, 1.14, *P* = 0.008). Compared with groups of retinal age gap within ± 3 years, retinal age gap less than minus 3 years was associated with a 9% decreased multimorbidity risk (model 1: HR = 0.91, CI 0.83, 0.99, *P* = 0.038) while retinal age gap more than 3 years showed an 15% increased risk of multimorbidity incidence (model 1: HR = 1.15, CI 1.06, 1.25, *P* = 0.001). Individuals with a retinal age gap of more than 3 years showed 12% increased risk of multimorbidity incidence in fully adjusted models. In addition, the Kaplan-Meier survival curves for each retinal age gap group did not cross, supporting the proportional hazards assumption (Supplementary Figure 2).

**Table 4:** Association between retinal age gap and incident multimorbidity

Retinal age gap	Model 1		Model 2		Model 3	
	HR (95%CI)	*p* value	HR (95%CI)	*p* value	HR (95%CI)	*p* value
Retinal age gap, per 5 years	1.12 (1.07, 1.18)	**<0.001**	1.09 (1.03, 1.15)	**0.003**	1.08 (1.02, 1.14)	**0.008**
Retinal age gap group						
> 3 years younger	0.91 (0.83, 0.99)	**0.038**	0.94 (0.85, 1.03)	0.195	0.94 (0.85, 1.04)	0.258
± 3 years	Reference	**-**	Reference	**-**	Reference	**-**
> 3 years older	1.15 (1.06, 1.25)	**0.001**	1.13 (1.02, 1.24)	**0.015**	1.12 (1.02, 1.24)	**0.019**

*HR* hazard ratio, *CI* confident interval

Model 1 adjusted for age and sex

Model 2 adjusted for covariates in model 1 and ethnicity, Townsend, education, physical activity, smoking status, alcohol drinking status, body mass index

Model 3 adjusted for covariates in model 2 and longevity genetic risk scores (GRS)

**Fig. 1:**
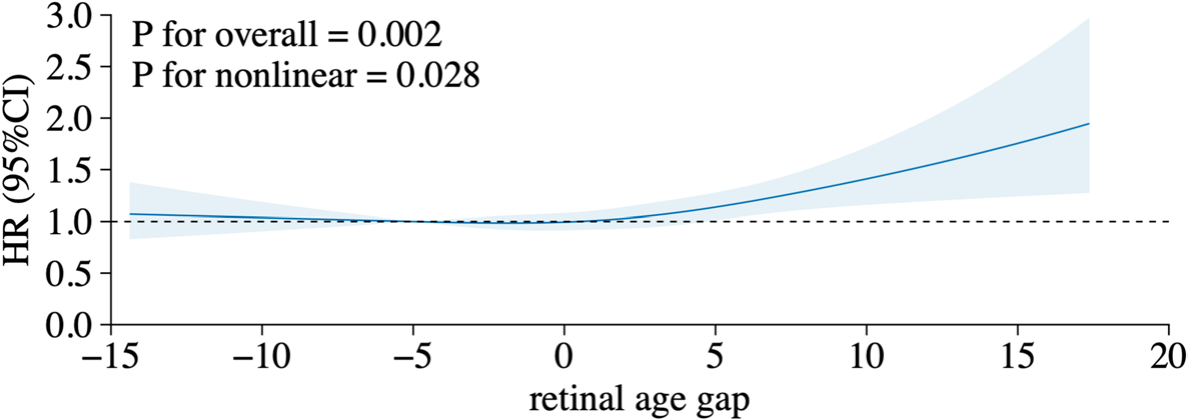
Nonlinear association between retinal age gap and incident multimorbidity. The model was fitted with a restricted cubic spline for retinal age gap adjusted for age, sex, ethnicity, Townsend, education, physical activity, smoking status, alcohol drinking status, body mass index, and longevity genetic risk scores (GRS). Evidence of an overall and nonlinear association between retinal age gap and multimorbidity risk was observed (*P*_overall_ = 0.002; *P*_nonlinear_ = 0.028). The association between retinal age gaps and multimorbidity is depicted as a *J*-shaped curve, where positive retinal age gaps were associated with substantially increased risks of multimorbidity

Restricted cubic spline analyses showed that the risk of incident multimorbidity increased significantly when the retinal age gap reached −1.78 years (*P*-overall = 0.002; *P*-nonlinear = 0.028, Fig. 1). After adding interactive terms in the cox model, we identified significant interaction effects between retinal age gap and smoking status (*p* = 0.017) as well as education level (*p* < 0.001).

## Discussion

In this prospective large-scale population-based study, we demonstrated that each 5-year increase of retinal age gap was independently associated with an 8% greater risk of incident multimorbidity. Retinal age more than 3 years older from normal ageing indicated a 12% increased risk of multimorbidity compared to those having retinal age gap within ±3 years. These findings suggest retinal age gap is a promising biomarker of future occurrence of multimorbidity independent of traditional risk factors.

To the best of our knowledge, this is the first study to assess the association between biological age and multimorbidity. We utilised retinal age, which possesses many advantages over most of existing aging biomarkers, including its non-invasive nature, rapid assessment, and high accuracy. Specifically, retinal images take up to 5 min per scan and the DL model can calculate the age gap within seconds. Retinal age achieved a MAE of 3.02 years which outperformed omics clocks (e.g., epigenetic clock: 3.3–5.2 years [9, 24], transcriptome age: 6.2–7.8 years [25, 26]) and other imaging-based clocks (e.g., brain age: 4.3–7.3 years) [27, 28].

Although the biological mechanisms underlying the association between retinal age gap and multimorbidity have not been fully established, several hypotheses have been proposed. Individuals with higher retinal age gaps may exhibit a higher prevalence of harmful lifestyle behaviours. For example, previous studies have shown a correlation between retinal age and lifestyle factors such as smoking and physical inactivity [14, 29, 30]. Additionally, the retinal vessels linked to the systemic circulation can undergo similar pathological changes responding to ageing risk factors [31]. Several hallmarks such as oxidative stress, chronic inflammation, and DNA damage could be induced during ageing process [32], leading to alterations in vascular dysfunction as reflected by the retinal microvasculature [33, 34]. Consistent with our findings, microvascular areas—particularly those surrounding visible vessels in fundus imaging—were highlighted as key features for age estimation. The retinal age gap may capture these alterations, acting as an early indicator of systemic aging and end-organ damage. These changes could serve as early signs of multimorbidity before its onset. Thus, multimorbidity can be understood as a consequence of systemic ageing, with retinal age providing a quantifiable marker of this process. Further research is needed to explore the underlying biological mechanisms.

Our findings have several important clinical implications. An increased retinal age gap is a promising biomarker for predicting the future occurrence of multimorbidity. Despite the increasing prevalence of patients with multimorbidity in the ageing society, clinical practice guidelines remain primarily built around single diseases. However, as opposed to those with only a single disease, people with multimorbidity experience a poorer quality of life and are higher users of ambulatory and inpatient care [35, 36], which can lead to many undesirable effects [37, 38]. By recognizing deviations from normal ageing, we can identify individuals at higher risk of developing multimorbidity. This early identification facilitates patients’ self-management and personalized interventions before disease onset. The potential for proactive, personalized healthcare based on retinal age gap assessment represents a step forward in addressing the complex needs of an ageing society.

Our study benefits from clear strengths such as the large sample size and multi-centre study design, extensive adjustments for covariates and long follow-up period. However, our findings should be interpreted cautiously considering its limitations. Firstly, while large, the UK Biobank cohort was mostly composed of relatively young and healthy Caucasian adults, lending itself to selection bias, which may limit generalizability [39]. Secondly, due to the observational study design, we could not infer causation. Thirdly, due to the lack of longitudinal data of fundus images, we could not explore the association of dynamic changes of retinal age gap with incident multimorbidity. Moreover, the possibility of residual confounding cannot be fully excluded. Furthermore, while our analysis focused on the overall association between retinal age gap and multimorbidity, future research could explore whether this relationship differs across disease types, providing deeper insights into the biological mechanisms linking retinal ageing to specific health outcomes. Lastly, while the retinal age gap demonstrates strong correlative value in associating with systemic morbidity, its utility as a predictive biomarker warrants further exploration. By leveraging existing data, predictive modelling approaches comparing with established risk factors could assess whether the retinal age gap enhances forecasting capabilities for disease onset or progression. External validation in diverse populations could also confirm the robustness. Additionally, biological links between retinal changes and systemic aging would strengthen its applicability in clinical settings. These steps could transform the retinal age gap from a descriptive metric into a prognostic tool with actionable insights for personalized medicine.

In conclusion, our study suggests that retinal age gap is a promising biomarker of future occurrence of multimorbidity, independent of traditional risk factors. This can help identify individuals at higher risks of developing multimorbidity and facilitate patients’ self-management and personalized interventions before disease onset. Further research is needed to explore the underlying biological mechanisms.

## Supplementary Information

Below is the link to the electronic supplementary material.

Supplementary file1 (DOCX 382 KB)

## Data Availability

All data and materials should be accessed from UK Biobank via reasonable request.

## References

[1] Almirall J, Fortin M. The coexistence of terms to describe the presence of multiple concurrent diseases. J Comorb. 2013;3:4–9. 29090140 10.15256/joc.2013.3.22 PMC5636023

[2] Violan C, Foguet-Boreu Q, Flores-Mateo G, et al. Prevalence, determinants and patterns of multimorbidity in primary care: a systematic review of observational studies. PLoS One. 2014;9(7):e102149. 25048354 10.1371/journal.pone.0102149 PMC4105594

[3] United Nations, Department of economic and social affairs, population division. World population ageing 2017, 2017. Accessed 10 June 2024. https://www.un.org/en/development/desa/population/publications/pdf/ageing/WorldPopulationAgeing2019-Highlights.pdf.

[4] Jylhava J, Pedersen NL, Hagg S. Biological AGE PREDICTors. EBioMedicine. 2017;21:29–36. 28396265 10.1016/j.ebiom.2017.03.046 PMC5514388

[5] Duan R, Fu Q, Sun Y, Li Q. Epigenetic clock: a promising biomarker and practical tool in aging. Ageing Res Rev. 2022;81:101743. 36206857 10.1016/j.arr.2022.101743

[6] Vaiserman A, Krasnienkov D. Telomere length as a marker of biological age: state-of-the-art, open issues, and future perspectives. Front Genet. 2020;11:630186. 33552142 10.3389/fgene.2020.630186 PMC7859450

[7] Cole JH, Franke K. Predicting age using neuroimaging: innovative brain ageing biomarkers. Trends Neurosci. 2017;40(12):681–90. 29074032 10.1016/j.tins.2017.10.001

[8] Zhu Z, Shi D, Guankai P, et al. Retinal age gap as a predictive biomarker for mortality risk. Br J Ophthalmol. 2023;107(4):547–54. 35042683 10.1136/bjophthalmol-2021-319807

[9] Yu Z, Chen R, Gui P, et al. Retinal age estimation with temporal fundus images enhanced progressive label distribution learning. paper presented at: medical image computing and computer assisted intervention – MICCAI 2023; 2023//, 2023; Cham.

[10] Flammer J, Konieczka K, Bruno RM, Virdis A, Flammer AJ, Taddei S. The eye and the heart. Eur Heart J. 2013;34(17):1270–8. 23401492 10.1093/eurheartj/eht023 PMC3640200

[11] London A, Benhar I, Schwartz M. The retina as a window to the brain-from eye research to CNS disorders. Nat Rev Neurol. 2013;9(1):44–53. 23165340 10.1038/nrneurol.2012.227

[12] Zhu Z, Chen Y, Wang W, et al. Association of retinal age gap with arterial stiffness and incident cardiovascular disease. Stroke. 2022;53(11):3320–8. 35880520 10.1161/STROKEAHA.122.038809

[13] Zhu Z, Hu W, Chen R, et al. Retinal age gap as a predictive biomarker of stroke risk. BMC Med. 2022;20(1):466. 36447293 10.1186/s12916-022-02620-w PMC9710167

[14] Zhu Z, Liu D, Chen R, et al. The association of retinal age gap with metabolic syndrome and inflammation. J Diabetes. 2023;15(3):237–45. 36919192 10.1111/1753-0407.13364 PMC10036256

[15] Hu W, Wang W, Wang Y, et al. Retinal age gap as a predictive biomarker of future risk of Parkinson’s disease. Age Ageing. 2022;51(3). 10.1093/ageing/afac062 10.1093/ageing/afac062 PMC8966015 35352798

[16] Zhang S, Chen R, Wang Y, et al. Association of retinal age gap and risk of kidney failure: a UK biobank study. Am J Kidney Dis. 2023;81(5):537-544.e531. 10.1053/j.ajkd.2022.09.018 36481699

[17] Skou ST, Mair FS, Fortin M, et al. Multimorbidity. Nat Rev Dis Primers. 2022;8(1):48. 35835758 10.1038/s41572-022-00376-4 PMC7613517

[18] Yarnall AJ, Sayer AA, Clegg A, Rockwood K, Parker S, Hindle JV. New horizons in multimorbidity in older adults. Age Ageing. 2017;46(6):882–8. 28985248 10.1093/ageing/afx150 PMC5860018

[19] Sudlow C, Gallacher J, Allen N, et al. UK biobank: an open access resource for identifying the causes of a wide range of complex diseases of middle and old age. PLoS Med. 2015;12(3):e1001779. 25826379 10.1371/journal.pmed.1001779 PMC4380465

[20] Chua SYL, Thomas D, Allen N, et al. Cohort profile: design and methods in the eye and vision consortium of UK Biobank. BMJ Open. 2019;9(2):e025077. 30796124 10.1136/bmjopen-2018-025077 PMC6398663

[21] Fu H, Wang B, Shen J, et al. Evaluation of retinal image quality assessment networks in different color-spaces. Paper presented at: Medical Image Computing and Computer Assisted Intervention–MICCAI 2019: 22nd International Conference, Shenzhen, China, October 13–17, 2019, Proceedings, Part I 222019

[22] Adams J, Ryan V, White M. How accurate are Townsend deprivation scores as predictors of self-reported health? A comparison with individual level data. J Public Health (Oxf). 2005;27(1):101–6. 15564276 10.1093/pubmed/fdh193

[23] Timmers P, Wilson JF, Joshi PK, Deelen J. Multivariate genomic scan implicates novel loci and haem metabolism in human ageing. Nat Commun. 2020;11(1):3570. 32678081 10.1038/s41467-020-17312-3 PMC7366647

[24] Weidner CI, Lin Q, Koch CM, et al. Aging of blood can be tracked by DNA methylation changes at just three CpG sites. Genome Biol. 2014;15(2):R24. 24490752 10.1186/gb-2014-15-2-r24 PMC4053864

[25] Fleischer JG, Schulte R, Tsai HH, et al. Predicting age from the transcriptome of human dermal fibroblasts. Genome Biol. 2018;19(1):221. 30567591 10.1186/s13059-018-1599-6 PMC6300908

[26] Peters MJ, Joehanes R, Pilling LC, et al. The transcriptional landscape of age in human peripheral blood. Nat Commun. 2015;6:8570. 26490707 10.1038/ncomms9570 PMC4639797

[27] Liem F, Varoquaux G, Kynast J, et al. Predicting brain-age from multimodal imaging data captures cognitive impairment. Neuroimage. 2017;148:179–88. 27890805 10.1016/j.neuroimage.2016.11.005

[28] Cole JH, Ritchie SJ, Bastin ME, et al. Brain age predicts mortality. Mol Psychiatry. 2018;23(5):1385–92. 28439103 10.1038/mp.2017.62 PMC5984097

[29] Chen R, Zhang J, Shang X, Wang W, He M, Zhu Z. Central obesity and its association with retinal age gap: insights from the UK Biobank study. Int J Obes (Lond). 2023;47(10):979–85. 37491535 10.1038/s41366-023-01345-x PMC10511312

[30] Chen R, Xu J, Shang X, et al. Association between cardiovascular health metrics and retinal ageing. Geroscience. 2023;45(3):1511–21. 36930331 10.1007/s11357-023-00743-3 PMC10400488

[31] Patton N, Aslam T, Macgillivray T, Pattie A, Deary IJ, Dhillon B. Retinal vascular image analysis as a potential screening tool for cerebrovascular disease: a rationale based on homology between cerebral and retinal microvasculatures. J Anat. 2005;206(4):319–48. 15817102 10.1111/j.1469-7580.2005.00395.x PMC1571489

[32] Lopez-Otin C, Blasco MA, Partridge L, Serrano M, Kroemer G. The hallmarks of aging. Cell. 2013;153(6):1194–217. 23746838 10.1016/j.cell.2013.05.039 PMC3836174

[33] Gao H, Hollyfield JG. Aging of the human retina. Differential loss of neurons and retinal pigment epithelial cells. Invest Ophthalmol Vis Sci. 1992;33(1):1-17. 1730530

[34] Panda-Jonas S, Jonas JB, Jakobczyk-Zmija M. Retinal photoreceptor density decreases with age. Ophthalmology. 1995;102(12):1853–9. 9098287 10.1016/s0161-6420(95)30784-1

[35] Gijsen R, Hoeymans N, Schellevis FG, Ruwaard D, Satariano WA, van den Bos GA. Causes and consequences of comorbidity: a review. J Clin Epidemiol. 2001;54(7):661–74. 11438406 10.1016/s0895-4356(00)00363-2

[36] Fortin M, Lapointe L, Hudon C, Vanasse A, Ntetu AL, Maltais D. Multimorbidity and quality of life in primary care: a systematic review. Health Qual Life Outcomes. 2004;2:51. 15380021 10.1186/1477-7525-2-51 PMC526383

[37] Uhlig K, Leff B, Kent D, et al. A framework for crafting clinical practice guidelines that are relevant to the care and management of people with multimorbidity. J Gen Intern Med. 2014;29(4):670–9. 24442332 10.1007/s11606-013-2659-y PMC3965742

[38] Boyd CM, Darer J, Boult C, Fried LP, Boult L, Wu AW. Clinical practice guidelines and quality of care for older patients with multiple comorbid diseases: implications for pay for performance. JAMA. 2005;294(6):716–24. 16091574 10.1001/jama.294.6.716

[39] Fry A, Littlejohns TJ, Sudlow C, et al. Comparison of sociodemographic and health-related characteristics of UK biobank participants with those of the general population. Am J Epidemiol. 2017;186(9):1026–34. 28641372 10.1093/aje/kwx246 PMC5860371

