# Supplementary material for: Accelerated retinal ageing and multimorbidity in middle-aged and older adults
Source: GeroScience. 2025 Mar 4;47(3):4291–300. doi: 10.1007/s11357-025-01581-1 (PMC12181587; doi:10.1007/s11357-025-01581-1)
Supplement: Supplementary file 1 — Supplementary file1 (DOCX 382 KB) [file 11357_2025_1581_MOESM1_ESM.docx]

Supplementary Materials

Supplementary table 1. Field codes for diseases of interest

Supplementary table 2. ICD codes for diseases of interest

Supplementary Table 3. Regression analysis between baseline characteristic and retinal age gaps.

Supplementary Figure 1. Distribution of Retinal age gap in studied population.

Supplementary Figure 2. Adjusted Kaplan-Meier survival curves for each group of retinal age gap.

Supplementary table 1. Field codes for diseases of interest

| **Long term condition grouping** | **Conditions included as reported by participants** | **Field Code** |
| --- | --- | --- |
| High cholesterol | High cholesterol | 1473 |
| Hypertension | Hypertension | 1065 |
|  | Essential Hypertension | 1072 |
| Atrial Fibrillation | Atrial Fibrillation | 1471 |
| Coronary heart disease | Heart attack/Myocardial Infarction | 1075 |
|  | Angina | 1074 |
| Heart failure | Cardiomyopathy | 1079 |
|  | Hypertrophic cardiomyopathy | 1588 |
|  | Heart failure/pulmonary oedema | 1076 |
| Stroke/Transient Ischaemic Attack (TIA) | Stroke | 1081 |
|  | TIA | 1082 |
|  | Subarachnoid haemorrhage | 1083 |
|  | Brain haemorrhage | 1086 |
|  | Ischaemic stroke | 1583 |
| Peripheral vascular disease | Peripheral vascular disease | 1067 |
|  | Leg claudication/intermittent claudication | 1087 |
| Diabetes | Diabetic nephropathy | 1607 |
|  | Diabetic neuropathy/ulcers | 1468 |
|  | Diabetes | 1220 |
|  | Type 1 diabetes | 1222 |
|  | Type 2 diabetes | 1223 |
|  | Diabetic eye disease | 1276 |
| Chronic Obstructive Pulmonary Disease (COPD) | COPD/chronic obstructive airways disease | 1112 |
|  | Emphysema/chronic bronchitis | 1113 |
|  | Emphysema | 1472 |
| Bronchiectasis | Bronchiectasis | 1114 |
| Parkinson’s disease | Parkinson’s disease | 1262 |
| Multiple sclerosis | Multiple Sclerosis | 1261 |
| Dementia | Dementia, Alzheimer’s disease, Cognitive impairment | 1263 |
| Dyspepsia | Gastro-oesophageal reflux (GORD)/gastric reflux | 1138 |
|  | Oesophagitis /Barrett's oesophagus | 1139 |
|  | Gastric stomach ulcers | 1142 |
|  | Gastric erosions/gastritis | 1143 |
|  | Duodenal ulcer | 1457 |
|  | Dyspepsia/indigestion | 1510 |
|  | Hiatus hernia | 1474 |
|  | Helicobacter pylori | 1442 |
| Diverticular disease | Diverticular disease | 1458 |
|  | Diverticulitis | 1458 |
| Treated constipation | Constipation | 1599 |
| Osteoporosis | Osteoporosis | 1465 |
| Fracture | fracture pelvis | 1647 |
|  | fracture neck of femur / hip | 1648 |
|  | fracture patella / knee | 1650 |
| Chronic kidney disease | Polycystic kidney | 1427 |
|  | Diabetic nephropathy | 1607 |
|  | Renal/kidney failure | 1192 |
|  | Renal failure requiring dialysis | 1193 |
|  | Renal failure not requiring dialysis | 1194 |
|  | Kidney nephropathy | 1519 |
|  | Immunoglobulin A (IgA) nephropathy | 1520 |
| Chronic liver disease | Oesophageal varices | 1141 |
|  | Non infective hepatitis | 1157 |
|  | Liver failure/cirrhosis | 1158 |
|  | Primary biliary cirrhosis | 1506 |
| Glaucoma | Glaucoma | 1277 |
| Cataract | Cataract | 1278 |
| AMD | AMD | 1528 |
|  | Lung cancer | 1001 |
| Cancer | Skin Cancer Non-melanoma | 1060 |
|  | Melanoma | 1059 |
|  | Stomach Cancer | 1018 |
|  | Oesophageal cancer | 1017 |
|  | Colon cancer | 1022 |
|  | Prostate cancer | 1044 |
|  | Ovarian cancer | 1039 |
|  | Breast cancer | 1002 |
|  | Cancer of lip/mouth/pharynx/oral cavity | 1004 |
|  | Salivary gland cancer | 1005 |
|  | Larynx/throat cancer | 1006 |
|  | Nasal cavity cancer | 1007 |
|  | Ear cancer | 1008 |
|  | Sinus cancer | 1009 |
|  | Lip cancer | 1010 |
|  | Tongue cancer | 1011 |
|  | Gum cancer | 1012 |
|  | Parotid gland cancer | 1015 |
|  | Other salivary gland cancer | 1016 |
|  | Small intestine/small bowel cancer | 1019 |
|  | Large bowel cancer/colorectal cancer | 1020 |
|  | Anal cancer | 1021 |
|  | Liver/hepatocellular cancer | 1024 |
|  | Gallbladder/bile duct cancer | 1025 |
|  | Pancreas cancer | 1026 |
|  | Small cell lung cancer | 1027 |
|  | Non-small cell lung cancer | 1028 |
|  | Peripheral nerve/autonomic nerve cancer | 1029 |
|  | Eye and/or adnexal cancer | 1030 |
|  | Meningeal cancer / malignant meningioma | 1031 |
|  | Brain cancer / primary malignant brain tumour | 1032 |
|  | Spinal cord or cranial nerve cancer | 1033 |
|  | Kidney/renal cell cancer | 1034 |
|  | Bladder cancer | 1035 |
|  | Other cancer of urinary tract | 1036 |
|  | Female genital tract cancer | 1037 |
|  | Male genital tract cancer | 1038 |
|  | Cervical cancer | 1041 |
|  | Vaginal cancer | 1042 |
|  | Vulval cancer | 1043 |
|  | Testicular cancer | 1045 |
|  | Penis cancer | 1046 |
|  | Lymphoma | 1047 |
|  | Leukaemia | 1048 |
|  | Multiple myeloma | 1050 |
|  | Myelofibrosis or myelodysplasia | 1051 |
|  | Hodgkins lymphoma / hodgkins disease | 1052 |
|  | Non-hodgkins lymphoma | 1053 |
|  | Chronic lymphocytic | 1055 |
|  | Chronic myeloid | 1056 |
|  | Other haematological malignancy | 1058 |
|  | Basal cell carcinoma | 1061 |
|  | Squamous cell carcinoma | 1062 |
|  | Primary bone cancer | 1063 |
|  | Mesothelioma | 1064 |
|  | Thyroid cancer | 1065 |
|  | Parathyroid cancer | 1066 |
|  | Adrenal cancer | 1067 |
|  | Sarcoma/fibrosarcoma | 1068 |
|  | Malignant lymph node, unspecified | 1070 |
|  | Metastatic cancer (unknown primary) | 1071 |
|  | CIN/pre-cancer cells cervix | 1072 |
|  | Rodent ulcer | 1073 |
|  | Acute myeloid leukaemia | 1074 |
|  | Retinoblastoma | 1075 |
|  | Kaposi's sarcoma | 1076 |
|  | Mouth cancer | 1077 |
|  | Tonsil cancer | 1078 |
|  | Oropharynx / oropharyngeal cancer | 1079 |
|  | Trachea cancer | 1080 |
|  | Thymus cancer / malignant thymoma | 1081 |
|  | Heart / mediastinum cancer | 1082 |
|  | Respiratory / intrathoracic cancer | 1084 |
|  | Bone metastases / bony secondaries | 1085 |
|  | Appendix cancer | 1086 |
|  | Fallopian tube cancer | 1087 |
|  | Malignant insulinoma | 1088 |

Supplementary table 2. ICD codes for diseases of interest

| **Long term condition grouping** | **Conditions included as reported by participants** | **ICD10 code** | **ICD9 code** |
| --- | --- | --- | --- |
| High cholesterol | Disorders of lipoprotein metabolism and other lipidaemias | E78 | 2720 |
| Hypertension | Essential (primary) hypertension | I10 | 401 |
|  | Hypertensive heart disease | I11 | 402 |
|  | Hypertensive heart disease with (congestive) heart failure | I110 | 403 |
|  | Hypertensive heart disease without (congestive) heart failure | I119 | 404 |
|  | Hypertensive renal disease | I12 | 405 |
|  | Hypertensive renal disease with renal failure | I120 |  |
|  | Hypertensive renal disease without renal failure | I129 |  |
|  | Hypertensive heart and renal disease | I13 |  |
|  | Hypertensive heart and renal disease with (congestive) heart failure | I130 |  |
|  | Hypertensive heart and renal disease with renal failure | I131 |  |
|  | Hypertensive heart and renal disease with both (congestive) heart failure and renal failure | I132 |  |
|  | Hypertensive heart and renal disease, unspecified | I139 |  |
|  | Secondary hypertension | I15 |  |
|  | Renovascular hypertension | I150 |  |
|  | Hypertension secondary to other renal disorders | I151 |  |
|  | Hypertension secondary to endocrine disorders | I152 |  |
|  | Other secondary hypertension | I158 |  |
|  | Secondary hypertension, unspecified | I159 |  |
| Atrial Fibrillation | Atrial fibrillation and flutter | I48 | 427 |
|  | Paroxysmal atrial fibrillation | I480 |  |
|  | Persistent atrial fibrillation | I481 |  |
|  | Chronic atrial fibrillation | I482 |  |
|  | Atrial fibrillation and atrial flutter, unspecified | I489 |  |
| Coronary Heart Disease | Angina pectoris | I20 | 413 |
|  | Acute myocardial infarction | I21 | 410 |
|  | Subsequent myocardial infarction | I22 | 411 |
|  | Certain current complications following acute myocardial infarction | I23 | 412 |
|  | Other acute ischaemic heart diseases | I24 | 414 |
|  | Chronic ischaemic heart disease | I25 |  |
| Heart failure | Cardiomyopathy | I42 | 425 |
|  | Heart failure | I50 | 428 |
| Stroke/Transient Ischaemic Attack (TIA) | Stroke, not specified as haemorrhage or infarction | I64 | 438 |
|  | Occlusion and stenosis of precerebral arteries, not resulting in cerebral infarction | I65 | 435 |
|  | Subarachnoid haemorrhage | I60 | 430 |
|  | Intracerebral haemorrhage | I61 | 431 |
|  | Other nontraumatic intracranial haemorrhage | I62 | 432 |
|  | Occlusion and stenosis of cerebral arteries, not resulting in cerebral infarction | I66 | 433 |
|  | Cerebral infarction | I63 | 434 |
|  | Acute but ill-defined cerebrovascular disease |  | 436 |
|  | Other and ill-defined cerebrovascular disease |  | 437 |
| Peripheral vascular disease | Other aneurysm | I72 | 440 |
|  | Other peripheral vascular diseases | I73 | 443 |
|  | Arterial embolism and thrombosis |  | 444 |
| Diabetes | Diabetic polyneuropathy | G632 | 250 |
|  | Diabetic mononeuropathy | G590 |  |
|  | Diabetic retinopathy | H360 |  |
|  | Diabetic cataract | H280 |  |
|  | Insulin-dependent diabetes mellitus | E10 |  |
|  | Non-insulin-dependent diabetes mellitus | E11 |  |
|  | Malnutrition-related diabetes mellitus | E12 |  |
|  | Other specified diabetes mellitus | E13 |  |
|  | Unspecified diabetes mellitus | E14 |  |
|  | Congenital iodine-deficiency syndrome | E00 | 240 |
| Chronic obstructive pulmonary disease (COPD) | Simple and mucopurulent chronic bronchitis | J41 | 491 |
|  | Unspecified chronic bronchitis | J42 |  |
|  | Emphysema | J43 | 492 |
|  | Other chronic obstructive pulmonary disease | J44 | 494 |
| Bronchiectasis | Bronchiectasis | J47 | 494 |
| Parkinson’s disease | Parkinson's disease | G20 | 332 |
|  | Secondary Parkinsonism | G21 | 3321 |
|  | Parkinsonism in diseases classified elsewhere | G22 | 333 |
|  | Other degenerative diseases of basal ganglia | G23 |  |
|  | Extrapyramidal and movement disorder, unspecified | G259 |  |
|  | Extrapyramidal and movement disorders in diseases classified elsewhere | G26 |  |
|  | Multisystem degeneration | G903 |  |
| Multiple Sclerosis | Multiple sclerosis | G35 | 340 |
| Dementia | Creutzfeldt-Jakob disease | A810 | 290 |
|  | Dementia in Alzheimer's disease | F00 |  |
|  | Vascular dementia | F01 |  |
|  | Unspecified dementia | F03 |  |
|  | Delirium superimposed on dementia | F051 |  |
|  | Amnesic syndrome | F106 |  |
|  | Alzheimer's disease | G30 |  |
|  | Other degenerative diseases of nervous system, not elsewhere classified | G31 |  |
|  | Progressive vascular leukoencephalopathy | I673 |  |
| Dyspepsia | Gastro-oesophageal reflux disease | K21 | 53081 |
|  | Gastro-oesophageal reflux disease with oesophagitis | K210 | 5368 |
|  | Gastro-oesophageal reflux disease without oesophagitis | K219 |  |
|  | Oesophagitis | K20 |  |
|  | Barrett's oesophagus | K227 |  |
|  | Other specified diseases of oesophagus | K228 |  |
|  | Disease of oesophagus, unspecified | K229 |  |
|  | Disorders of oesophagus in diseases classified elsewhere | K23 |  |
|  | Gastric ulcer | K25 |  |
|  | Gastritis and duodenitis | K29 |  |
|  | Duodenal ulcer | K26 |  |
|  | Dyspepsia | K30 |  |
|  | Congenital hiatus hernia | Q401 |  |
|  | Helicobacter pylori [H.pylori] as the cause of diseases classified to other chapters | B980 |  |
| Diverticular disease | Diverticular disease of intestine | K57 | 562 |
| Treated constipation | Constipation | K590 | 5640 |
| Osteoporosis | Polyarthrosis | M15 | 7330 |
|  | Primary generalised (osteo)arthrosis | M150 |  |
|  | Primary generalized (osteo)arthrosis, Multiple sites | M1500 |  |
|  | Heberden's nodes (with arthropathy) | M151 |  |
|  | Coxarthrosis [arthrosis of hip] | M16 |  |
|  | Gonarthrosis [arthrosis of knee] | M17 |  |
|  |  |  |  |
| Fracture | Multiple fractures of lumbar spine and pelvis | S327 | 808 |
|  | Multiple fractures of lumbar spine and pelvis (closed) | S3270 |  |
|  | Fracture of femur | S72 | 820 |
|  | Fracture of patella | S820 | 8210 |
|  | Fracture of patella (closed) | S8200 | 824 |
| Chronic kidney disease | Polycystic kidney, infantile type | Q611 | 75315 |
|  | Polycystic kidney, adult type | Q612 |  |
|  | Polycystic kidney, unspecified | Q613 |  |
|  | Acute renal failure | N17 | 584 |
|  | Chronic renal failure | N18 | 585 |
|  | Unspecified renal failure | N19 | 586 |
|  | Renal complications | E112 | 587 |
|  | Other | N028 | 588 |
| Chronic Liver disease | Oesophageal varices | I85 | 571 |
|  | Toxoplasma hepatitis | B581 |  |
|  | Alcoholic hepatitis | K701 |  |
|  | Toxic liver disease with acute hepatitis | K712 |  |
|  | Toxic liver disease with chronic persistent hepatitis | K713 |  |
|  | Toxic liver disease with chronic lobular hepatitis | K714 |  |
|  | Toxic liver disease with chronic active hepatitis | K715 |  |
|  | Toxic liver disease with hepatitis, not elsewhere classified | K716 |  |
|  | Fibrosis and cirrhosis of liver | K74 |  |
|  | Primary biliary cirrhosis | K743 |  |
| Glaucoma | Glaucoma | H40 | 365 |
| Cataract | Senile cataract | H25 | 366 |
|  | Other cataract | H26 |  |
|  | Cataract and other disorders of lens in diseases classified elsewhere | H28 |  |
| AMD | Degeneration of macula and posterior pole | H353 | 3625 |
| Cancer | Lung Cancer | C34 | 162 |
|  | Non-melanoma skin cancer Other malignant neoplasms of skin | C44 | 173 |
|  | Mesothelioma | C45 | 174 |
|  | Melanoma Malignant melanoma of skin | C43 | 172 |
|  | Stomach Cancer Malignant neoplasm of stomach | C16 | 151 |
|  | Oesophageal cancer Malignant neoplasm of oesophagus | C15 | 150 |
|  | Colon cancer Malignant neoplasm of colon | C18 | 153 |
|  | Prostate cancer Malignant neoplasm of prostate | C61 | 185 |
|  | Ovarian cancer Malignant neoplasm of ovary | C56 | 183 |
|  | Breast cancer Malignant neoplasm of breast | C50 | 174 |
|  | Malignant neoplasm of lip | C00 | 140 |
|  | Malignant neoplasm of base of tongue | C01 | 141 |
|  | Malignant neoplasm of other and unspecified parts of tongue | C02 | 142 |
|  | Malignant neoplasm of gum | C03 | 143 |
|  | Malignant neoplasm of floor of mouth | C04 | 144 |
|  | Malignant neoplasm of palate | C05 | 145 |
|  | Malignant neoplasm of other and unspecified parts of mouth | C06 | 146 |
|  | Malignant neoplasm of parotid gland | C07 | 147 |
|  | Malignant neoplasm of other and unspecified major salivary glands | C08 | 148 |
|  | Malignant neoplasm of tonsil | C09 | 149 |
|  | Malignant neoplasm of oropharynx | C10 | 152 |
|  | Malignant neoplasm of nasopharynx | C11 | 155 |
|  | Malignant neoplasm of pyriform sinus | C12 | 156 |
|  | Malignant neoplasm of hypopharynx | C13 | 157 |
|  | Malignant neoplasm of other and ill-defined sites in the lip, oral cavity and pharynx | C14 | 158 |
|  | Malignant neoplasm of small intestine | C17 | 159 |
|  | Malignant neoplasm of rectosigmoid junction | C19 | 160 |
|  | Malignant neoplasm of anus and anal canal | C21 | 161 |
|  | Malignant neoplasm of liver and intrahepatic bile ducts | C22 | 163 |
|  | Malignant neoplasm of gallbladder | C23 | 164 |
|  | Malignant neoplasm of other and unspecified parts of biliary tract | C24 | 165 |
|  | Malignant neoplasm of pancreas | C25 | 166 |
|  | Malignant neoplasm of other and ill-defined digestive organs | C26 | 167 |
|  | Malignant neoplasm of nasal cavity and middle ear | C30 | 171 |
|  | Malignant neoplasm of accessory sinuses | C31 | 175 |
|  | Malignant neoplasm of larynx | C32 | 176 |
|  | Malignant neoplasm of trachea | C33 | 177 |
|  | Malignant neoplasm of thymus | C37 | 180 |
|  | Malignant neoplasm of heart, mediastinum and pleura | C38 | 181 |
|  | Malignant neoplasm of other and ill-defined sites in the respiratory system and intrathoracic organs | C39 | 184 |
|  | Malignant neoplasm of bone and articular cartilage of limbs | C40 | 186 |
|  | Malignant neoplasm of bone and articular cartilage of other and unspecified sites | C41 | 187 |
|  | Kaposi's sarcoma | C46 | 189 |
|  | Malignant neoplasm of peripheral nerves and autonomic nervous system | C47 | 190 |
|  | Malignant neoplasm of retroperitoneum and peritoneum | C48 | 191 |
|  | Malignant neoplasm of other connective and soft tissue | C49 | 192 |
|  | Malignant neoplasm of vulva | C51 | 193 |
|  | Malignant neoplasm of vagina | C52 | 194 |
|  | Malignant neoplasm of cervix uteri | C53 | 195 |
|  | Malignant neoplasm of corpus uteri | C54 | 196 |
|  | Malignant neoplasm of other and unspecified female genital organs | C57 | 197 |
|  | Malignant neoplasm of placenta | C58 | 198 |
|  | Malignant neoplasm of penis | C60 | 200 |
|  | Malignant neoplasm of testis | C62 | 201 |
|  | Malignant neoplasm of other and unspecified male genital organs | C63 | 202 |
|  | Malignant neoplasm of kidney, except renal pelvis | C64 | 203 |
|  | Malignant neoplasm of renal pelvis | C65 | 204 |
|  | Malignant neoplasm of ureter | C66 | 205 |
|  | Malignant neoplasm of bladder | C67 | 206 |
|  | Malignant neoplasm of other and unspecified urinary organs | C68 | 207 |
|  | Malignant neoplasm of eye and adnexa | C69 | 208 |
|  | Malignant neoplasm of meninges | C70 |  |
|  | Malignant neoplasm of brain | C71 |  |
|  | Malignant neoplasm of spinal cord, cranial nerves and other parts of central nervous system | C72 |  |
|  | Malignant neoplasm of thyroid gland | C73 |  |
|  | Malignant neoplasm of adrenal gland | C74 |  |
|  | Malignant neoplasm of other endocrine glands and related structures | C75 |  |
|  | Malignant neoplasm of other and ill-defined sites | C76 |  |
|  | Secondary and unspecified malignant neoplasm of lymph nodes | C77 |  |
|  | Secondary malignant neoplasm of respiratory and digestive organs | C78 |  |
|  | Secondary malignant neoplasm of other sites | C79 |  |
|  | Malignant neoplasm without specification of site | C80 |  |
|  | Hodgkin's disease | C81 |  |
|  | Follicular [nodular] non-Hodgkin's lymphoma | C82 |  |
|  | Diffuse non-Hodgkin's lymphoma | C83 |  |
|  | Peripheral and cutaneous T-cell lymphomas | C84 |  |
|  | Other and unspecified types of non-Hodgkin's lymphoma | C85 |  |
|  | Other specified types of T/NK-cell lymphoma | C86 |  |
|  | Malignant immunoproliferative diseases | C88 |  |
|  | Multiple myeloma and malignant plasma cell neoplasms | C90 |  |
|  | Lymphoid leukaemia | C91 |  |
|  | Myeloid leukaemia | C92 |  |
|  | Monocytic leukaemia | C93 |  |
|  | Other leukaemias of specified cell type | C94 |  |
|  | Leukaemia of unspecified cell type | C95 |  |
|  | Other and unspecified malignant neoplasms of lymphoid, haematopoietic and related tissue | C96 |  |
|  | Malignant neoplasms of independent (primary) multiple sites | C97 |  |

Supplementary Table 3. Regression analysis between baseline characteristic and retinal age gaps.

| Baseline Characteristic | Beta (95% CI) | *p* value |
| --- | --- | --- |
| Age, years | -0.241 (-0.245 to -0.237) | <0.001 |
| Sex (Male vs. Female) | -0.177 (-0.243 to -0.111) | <0.001 |
| Ethnicity (Others vs. White) | -0.921 (-1.039 to -0.803) | <0.001 |
| Townsend | 0.021 (0.010 to 0.032) | <0.001 |
| Education (Others vs. College/university) | -0.037 (-0.105 to 0.031) | 0.288 |
| Meeting moderate/vigorous/walking recommendation (Yes vs. No) | -0.184 (-0.279 to -0.089) | <0.001 |
| Smoking status (Ex/current vs. Never) | 0.395 (0.329 to 0.462) | <0.001 |
| Alcohol drinking status (Ex/current vs. Never) | 0.338 (0.181 to 0.495) | <0.001 |
| Body mass index, kg/m^2 | 0.026 (0.019 to 0.033) | <0.001 |
| Longevity genetic risk scores | -1.028 (-1.660 to -0.396) | 0.001 |
| Beta = regression coefficient; CI = confident interval. | |  |
| Model adjusted for age and sex. |  |  |


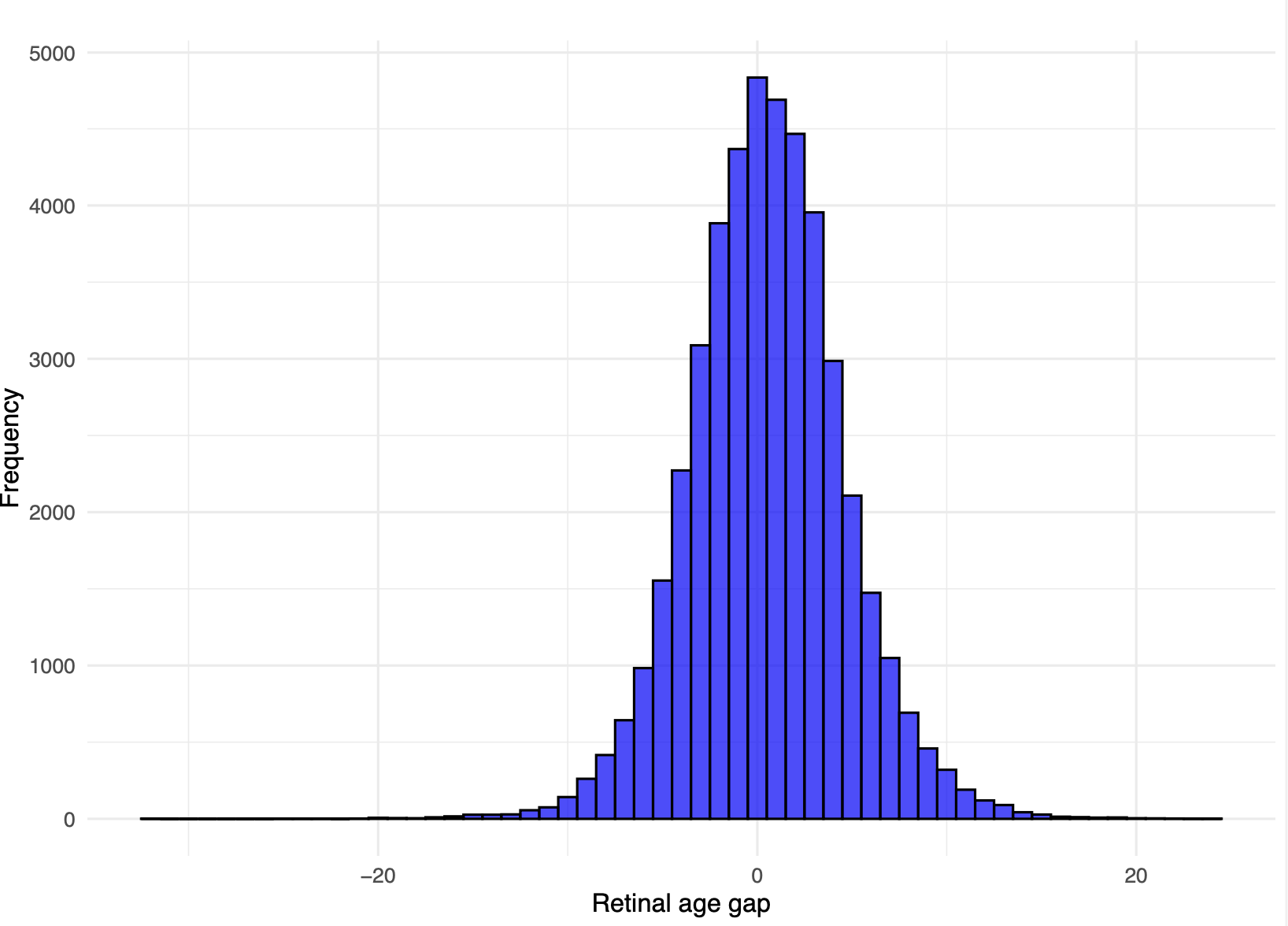


Supplementary Figure 1. Distribution of Retinal age gap in studied population. The distribution of the retinal age gap in the study population followed a nearly normal distribution.


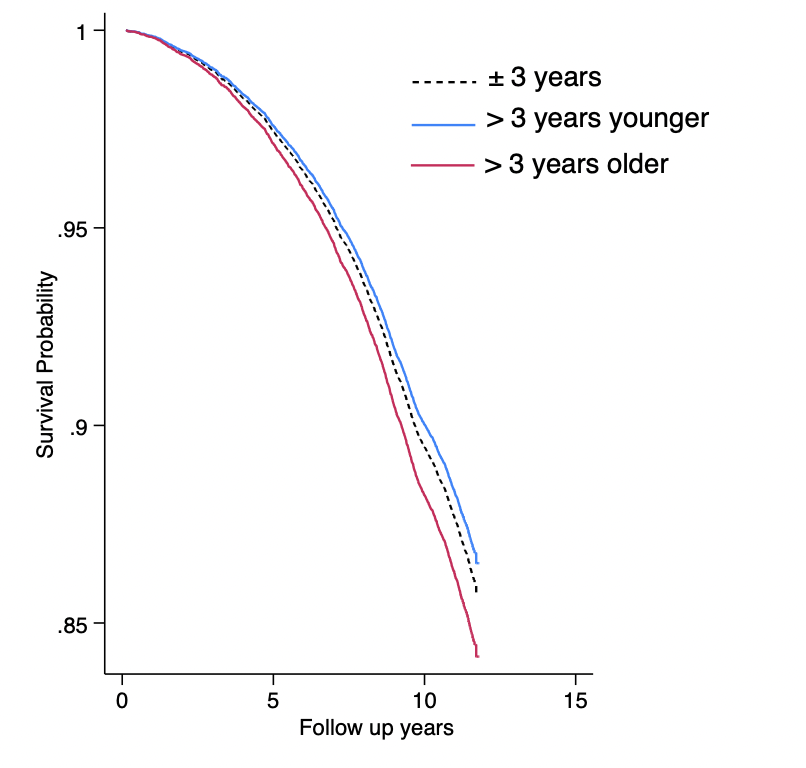


Supplementary Figure 2. Adjusted Kaplan-Meier survival curves for each group of retinal age gap. Multimorbidity risk is shown over time for participants in different retinal age gap groups. Participants were divided into three groups of patients who had a predicted retinal age > 3 years smaller than the chronological age (> 3 years younger), retinal age within a range of 3 years from their chronological age (within ± 3 years), and retinal age > 3 years greater than the chronological age (> 3 years older). Plots were based on Cox proportional hazards regression models, adjusted for age, sex, ethnicity, Townsend, education, physical activity, smoking status, alcohol drinking status, body mass index and Longevity genetic risk scores (GRS).
